# Aqueous Extract of *Allium sativum* (Linn.) Bulbs Ameliorated Pituitary-Testicular Injury and Dysfunction in Wistar Rats with Pb-Induced Reproductive Disturbances

**DOI:** 10.3889/oamjms.2016.039

**Published:** 2016-03-31

**Authors:** Abiodun O. Ayoka, Aderonke K. Ademoye, Christian E. Imafidon, Esther O. Ojo, Ayowole A. Oladele

**Affiliations:** 1 *Department of Physiological Sciences, Obafemi Awolowo University, Ile-Ife, Osun State, Nigeria*; 2 *Department of Medical Laboratory Science, College of Medicine, Afe Babalola University, Ado-Ekiti, Ekiti State, Nigeria*

**Keywords:** Lead Acetate, *Allium sativum* bulbs, Pituitary, Testis, Reproductive function, Rats

## Abstract

**AIM:**

To determine the effects of aqueous extract of Allium sativum bulbs (AEASAB) on pituitary-testicular injury and dysfunction in Wistar rats with lead-induced reproductive disturbances.

**MATERIALS AND METHODS:**

Male Wistar rats were divided into 7 groups such that the control group received propylene glycol at 0.2 ml/100 g intraperitoneally for 10 consecutive days, the toxic group received lead (Pb) alone at 15 mg/kg/day via intraperitoneal route for 10 days while the treatment groups were pretreated with lead as the toxic group after which they received graded doses of the extract at 50, 100 and 200 mg/kg/day via oral route for 28 days.

**RESULTS:**

Pb administration induced significant deleterious alterations in the antioxidant status of the brain and testis, sperm characterization (counts, motility and viability) as well as reproductive hormones (FSH, LH and testosterone) of exposed rats (p < 0.05). These were significantly reversed in the AEASAB-treated groups (p < 0.05). Also, there was marked improvement in the Pb-induced vascular congestion and cellular loss in the pituitary while the observed Pb-induced severe testicular vacuolation was significantly reversed in the representative photomicrographs, following administration of the extract.

**CONCLUSION:**

AEASAB treatment ameliorated the pituitary-testicular injury and dysfunction in Wistar rats with Pb-Induced reproductive disturbances.

## Introduction

Lead (Pb) is a heavy metal present in both organic (tetraethyl lead) and inorganic (lead acetate, lead chloride) forms in the environment [1]. Its unique properties which include high malleability, ductility, softness, low melting point and resistance to corrosion accounts for its widespread industrial uses such as in the production of electric storage batteries, ceramics, paints, plastics and automobiles [2, 3]. Its prolonged persistence in the environment results from its non-biodegradable nature [4, 5], thus making it a potent environmental and occupational toxin. It is reputed for its wide range of toxic physiological, biochemical and histological effects on the brain [6], kidney [7], liver [8], blood [9], the endocrine system [10] and testis [11,12] of exposed subjects. The Pb-induced oxidative stress in tissues, which leads to oxidative damage, is known to follow two pathways; first is the generation of reactive oxygen species (ROS) while the second pathway is the depletion of antioxidants reserve through the generation of ROS [13, 14]. If not treated in time, its toxic effect can be more severe and is usually characterized by lethargy, delirium, encephalopathy, vomiting, convulsions and coma [15, 16]. Even in the light of recent advancement in the field of scientific research, there seem to be no “safe” level of exposure to Pb neither has there been any report of a level that is positively beneficial in biological systems. Ethnobotanical approaches in ameliorating its toxic effect should become an area of scientific interest since established therapy, such as the use of dimercaptosuccinic acid (DMSA), are often burdened with undesirable side effects [17].

Until the nineteenth century, when synthetic drugs were developed herbs, remained the basis for almost all medicinal therapy [18]. In recent times, about 40% of prescriptions still contain herbs and because its use is often associated with lesser side effects, the interest for herbal remedies instead of chemical drugs is on the increase [19]. WHO, in 2008 reported that medicinal plants are used as a primary health care aid among 80% of the world’s population in the form of their active components or the plant extracts due to their health-boosting benefits [20]. These plants have gained relevance in the field of fertility study as there are reports of their role as modulators of spermatogenesis [21] as well as their effects on sperm quality [22].

*Allium sativum*, commonly called garlic, is a medicinal plant credited to have remarkable pharmacological properties; its active agent being allicin which imparts its characteristic odour [23]. Its antibiotic, antioxidant, anti-inflammatory, antimicrobial and antithrombotic potentials have been proven over different disease conditions such as prostate cancer, stroke [23, 24] to mention a few. Despite these remarkable medicinal benefits, there is a dearth of literature on its effects on Pb-induced pituitary-testicular injury.

The present study investigated the effects of aqueous extract of *Allium sativum* (Linn.) bulbs on the pituitary and testis of Wistar rats with a Pb-induced compromise to reproductive function.

## Materials and Methods

### 

#### Plant, Drug and Chemical Materials

Fresh bulbs of *Allium sativum* were obtained from a commercial supplier in Lagere market, Ile-Ife and certified by a Taxonomist in the Department of Botany, Obafemi Awolowo University (OAU), Ile-Ife; where a voucher specimen (IFE- 013429) was deposited.

Lead acetate salt was purchased from Guangzhou Fischer Chemical Co., Ltd, Guangdong, China while vitamin E was purchased from Sinopharm Xingsha Pharmaceutics (Xiamen City, Fujian Province, P. R. China). Propylene glycol was purchased from Biovision, Milpitas (CA, USA). Assay kits for hormone analyses that were carried out were purchased from Monobind Inc., Lake Forest CA 92630, USA (Accu-Bind Elisa Microwells).

#### Plant Extraction

The extraction process was a modification of Belgin *et al* [25]. The modification was such that the washed and peeled samples of garlic were not washed with liquid nitrogen before lyophilizing. The extraction process is as described as follows; Fresh bulbs of *Allium sativum* were washed, peeled and weighed. These were thereafter pulverized in distilled water with the aid of a Waring blender (Waring Commercial, Torrington, CT). The resulting mixture was subjected to constant shaking for 12 hours with the aid of an Electric Shaker. Afterwards, it was filtered under vacuum using Buchner funnel and Whatman number 2 Filter Paper (Whatman PLC, Middlesex, UK). The filtrate was concentrated under vacuum using a Rotary Evaporator (HahnShin Scientific, HS-2005-N) and freeze-dried in a Lyophilizer at -40 ºC (Ilshin Lab. Co. Ltd, Seoul, Republic of Korea). The yield obtained was kept in a desiccator until when needed. The percentage (%) yield of ASBAE was calculated as shown below [26]:

Percentage (%) Yield = (Yield of the Extract/Weight of Pulverized Bulbs) * 100

#### Detection and Quantification of Saponins

Qualitatively, the presence of saponins was determined by Froth test as described by Benmehdi *et al* [27]. This is described as follows; 2 g of the powdered leaves was introduced into a beaker containing 100 mL of distilled water. The mixture was boiled in a water bath and filtered. The filtrate was introduced into ten test tubes and made up to the following volumes (1, 2, … 5 mL) of the mother solution. Then the final volume was readjusted to 10 mL with distilled water. All tubes were vigorously shaken for 15 s; formation of froth indicated the presence of saponins. Thereafter, the presence of saponins was quantitatively screened for by the method of Obadoni and Ochuko [28].

#### Detection of Terpenoids

The presence of terpenoids was qualitatively screened for using the method described by Benmehdi *et al* [27]. This is stated as follows; 2 ml of acetic anhydride was added to 0.5 g of the extract with 2 mL H_2_SO_4_. The colour change to red, pink or violet colour indicates the presence of terpenoids.

#### Detection and Quantification of Tannins

This was qualitatively screened using the methods described by Halilu *et al* [29]. The following procedures were adopted; a) *Lead acetate test*: to 1 mL of the extract, 2 drops of lead acetate solution was added. A coloured precipitate indicated the presence of tannins. b) *Bromine water test:* the plant extract was treated with 3 drops of bromine water. Non-formation of coloured precipitate indicated the presence of hydrolysable tannins. Thereafter, tannin was quantitatively screened for by the method of Allen *et al* [30].

#### Detection and Quantification of Flavonoids

*Sodium hydroxide test*, as described by Halilu *et al* [29], was used to qualitatively determine the presence of flavonoids in the extract. This involved the following procedure; the extract was treated with few drops of sodium hydroxide solution. Formation of intense yellow colour, which became colourless on the addition of dilute acid, indicated the presence of flavonoids. The quantitative screening of flavonoids was determined by the method of Obadoni and Ochuko [28].

#### Detection and Quantification of Alkaloids

As described by the method of Halilu *et al* [29], the extract was dissolved in dilute hydrochloric acid and filtered. Thereafter, the filtrate was divided into 3 portions and the following reagents were used to test for the presence of alkaloids;

a) *Mayer’s Test:* The filtrate was treated with Mayer’s reagent (Potassium Mercuric Iodide). The formation of a yellow coloured precipitate indicated the presence of alkaloids.
b) *Wagner’s Test:* The filtrate was treated with Wagner’s reagent (Iodine in Potassium Iodide). The formation of brown/reddish precipitate indicated the presence of alkaloids.
c) *Dragendroff’s Test:* The filtrate was treated with Dragendroff’s reagent (solution of Potassium Bismuth Iodide). The formation of reddish precipitate indicated the presence of alkaloids. Thereafter, the presence of alkaloids was quantified by the method of Harborne [31].

#### Detection and Quantification of Cardiac Glycosides

Qualitative determination of cardiac glycol-sides was by Keller-Kiliani test as described by Anjali and Sheetal [32]. The procedure is described as follows; 5 ml of the extract was treated with 2 ml of glacial acetic acid containing one drop of ferric chloride solution. This was underlayed with 1 ml of concentrated H_2_SO_4_. A brown ring of the interface indicated a deoxy sugar characteristic of cardenolides. A violet ring may appear below the brown ring, while in the acetic acid layer, a greenish ring may form just gradually throughout the thin layer. Thereafter, cardiac glycosides were quantified by the method of Harborne [31].

#### Determination of Oral Lethal Dose (LD_50_) of AEASAB

The oral lethal dose (LD_50_) of AEASAB was determined by the method of Lorke [33] and as modified by Imafidon *et al* [34]. A total of 17 adult Wistar rats were used for this study. In the initial phase of the experiment, 9 rats were divided into 3 groups of 3 rats each and were treated with AEASAB at graded doses of 5, 50 and 500 mg/kg, orally. The rats were observed for 24 hours. In the second phase, 8 rats were divided into 4 groups of 2 rats each and were treated with AEASAB at 350, 700, 1400 and 2800 mg/kg, orally. They were also examined for 24 hours and the LD_50_ was determined using the formula;

LD_50_ = √ aX b

Where a= the least dose that killed a rat; and b= highest dose that did not kill any rat.

#### Preparation of Stock Solutions of AEASAB and Pb

The choice of therapeutic doses of AEASAB was guided by its predetermined oral LD50; these were taken to be ≤ 10% of oral LD_50_. Thus, doses of 50, 100 and 200 mg/kg of AEASAB were prepared as follows; 500 mg of AEASAB was dissolved in 20 ml of propylene glycol to prepare a stock solution of 50 mg/kg of AEASAB. Stock solutions of 100 and 200 mg of ASBAE were prepared by each dissolving 1 g and 2 g of AEASAB in 20 ml of propylene glycol, respectively. The rats received 0.2 ml/100g of AEASAB, orally. Samples were stored in a deep-freezer after use while fresh samples were prepared every 48 hours. Freshly prepared solutions of vitamin E was obtained by dissolving 500 mg of the soft capsule in 10 ml of propylene glycol and administered at 0.2 ml/100 g rat which is an equivalent of 100 mg/kg body weight. This choice of adopted dose was guided by existing references [35-37].

One hundred and fifty milligrams (150 mg) of lead acetate salt was dissolved in 20 ml of distilled water and was administered to the rats at 0.2 ml/100 g. Therefore, each rat received 15 mg/kg/day of Pb solution for ten consecutive days, via intraperitoneal route (i.p.).

#### Animal Management and Experimental Design

A total of thirty-five (35) male Wistar rats, weighing 150-180 g, were used for this study. They were purchased from the Animal Holdings of the College of Health Sciences, OAU, Ile-Ife, Osun State, Nigeria where the study was carried out. They were housed in plastic cages under natural light/dark cycle and allowed to have access to standard laboratory rat chow (Caps Feed PLC Osogbo, Nigeria) and water *ad libitum*. All experimental protocols were in strict compliance with the guidelines for animal research, as detailed in the NIH Guidelines for the Care and Use of Laboratory Animals (National Academy of Sciences and National Institutes of Health Publications, 2011) and approved by local Institutional Research Committee.

The rats were divided into seven (7) groups of 5 rats each as follows; Group 1 (Control group) received propylene glycol (0.2 ml per 100 g body weight) orally, throughout the 38 days study period. Group 2 (Pb) received Pb alone at 15 mg/kg/day via intraperitoneal route for 10 consecutive days. Group 3 (Pb + recovery) were pre- treated with Pb as group 2 and thereafter left untreated for a period of 28 days. Group 4 (Pb + vitamin E) were pre-treated with Pb as group 2, followed by vitamin E (100 mg/kg) for 28 days. Groups 5, 6 and 7 were also pre-treated with Pb as group 2 and thereafter received graded doses of AEASAB at 50, 100 and 200 mg/kg/day, respectively via oral route for 28 days. Twenty-four hours after last administration of Pb (in group 2), AEASAB (in groups 5, 6 and 7) and after the recovery period (in group 3), rats were euthanized and blood samples were collected by cardiac puncture into separate EDTA bottles. These were centrifuged at 4000 rpm for 15minutes at -4ºC, using cold centrifuge (Centurium Scientific, Model 8881). Plasma obtained was collected into separate plain bottles for the assessment of the reproductive hormones using appropriate kits and Enzyme-Linked Immuno Sorbent Assay (ELISA) technique. The procedure for hormone assays was as provided in the appropriate kits. The left caudal epididymis of each rat was carefully excised for sperm characterisation. The left testis and 1 g of the brain of each rat were kept in a cooler for homogenates preparation while the right testis and the pituitary of each rat were fixed in Bouin’s fluid and 10 % formal saline solution, respectively for histopathological examination using Hematoxylin- Eosin (H & E) staining technique. The experimental design is as depicted in Table 1.

**Table 1:** Dose Regimen and Experimental Design

GROUPS (n = 5)	10 Days Pb Administration (15 mg/kg) via Intraperitoneal Route	28 Days Oral Treatment/Recovery Period
1	PG	PG*
2	Pb*	__
3	Pb	RP
4	Pb	100 mg/kg Vitamin E
5	Pb	50 mg/kg AEASAB
6	Pb	100 mg/kg AEASAB
7	Pb	200 mg/kg AEASAB

PG = Propylene glycol; Pb = Lead; RP = Recovery Period; AEASAB = Aqueous Extract of *Allium sativum* Bulbs;

* = Point that rats were sacrificed;

Group 1 = Control; Group 2 = Pb only; Group 3 = Pb + Recovery Period; Group 4 = Pb + 100 mg/kg Vitamin E; Group 5 = Pb + 50 mg/kg AEASAB; Group 6 = Pb + 100 mg/kg AEASAB; Group 7 = Pb + 200 mg/kg AEASAB.

#### Measurement of Body and Organ Weights

Weekly body weight of the rats was determined with the aid of a digital weighing balance (Hanson, China) to assess weekly weight change while at the point of sacrifice, the organ weights were determined using a sensitive weighing balance (Camry, China).

#### Sperm Characterisation

Sperm fluid from the caudal epididymis was squeezed onto a microscope slide and epididymal sperm counts were made using haemocytometer and expressed as million/ml of suspension. Epididymal sperm motility was assessed by calculating motile spermatozoa per unit area and expressed as motility in percentage. Using Eosin-Nigrosin stain, the sperm viability was determined by preparing uniform smear spermatozoa on the slides by the method of Bloom [38] and as described by Raji *et al* [39].

#### Hormone Assays

Plasma levels of FSH, LH and testosterone were determined using the laboratory protocols as provided in the aforementioned appropriate kits by Enzyme-Linked ImmunoSorbent Assay (ELISA) technique.

#### Measurements of Antioxidant Enzymes

The testis (left) and brain of each rat was carefully excised and weighed. 10% homogenate in phosphate buffer (100 Mm) was prepared at pH of 7.4. Each tissue was homogenized with 10ml of sucrose solution (0.25 M) using Electric Homogenizer (SI601001). The homogenates were centrifuged at 3000 rpm for 20 minutes and the supernatant were collected for the assessment of the following markers of lipid peroxidation and oxidative stress;

#### Estimation of Reduced Glutathione (GSH)

GSH level was estimated by the method of Beutler and Kelly [40]. 1ml of the supernatant, obtained above, was added to 0.5 ml of Ellman’s reagent (10 mM). 2 ml of phosphate buffer (0.2 M, pH 8.0) was, thereafter, added. The yellow colour developed was read at 412 nm against blank containing 3.5 ml of phosphate buffer. A series of standards were also treated similarly. The amount of GSH was expressed in the mg/100g tissue.

#### Determination of Thiobabituric Acid Reactive Substances (TBARS)

The total amount of lipid peroxidation products was determined by the thiobarbituric acid (TBA) method which measures the malondialdehyde (MDA) reactive products according to the method of Ohkawa *et al* [41]. To 0.5 ml of the sample was added 0.5 ml of phosphate buffer (0.1 M, pH 8.0) and 0.5 ml of 24% TCA. The resulting mixture was incubated at room temperature for 10 min, followed by centrifugation at 2000 rpm for 20 min. To 1 ml of the supernatant was added 0.25 ml of 0.33% TBA in 20% acetic acid and the resulting mixture was boiled at 95°C for 1 hr. The resulting pink colour product was cooled and absorbance was read at 532 nm (Extinction coefficient of MDA, ε532 = 1.53 x 105 M^-1^ cm^-1^).

#### Histopathological Examination

Samples of pituitary and testis were dehydrated in graded alcohol and embedded in paraffin wax. Sections > 4 μm thick were stained with Hematoxylin-Eosin and viewed under a Leica DM750 Camera Microscope which was used to take the photomicrographs at a magnification of x 100.

#### Statistical Analysis

The results obtained were collated and expressed as Mean ± Standard Error of Mean (S.E.M) and subjected to one-way analysis of variance (ANOVA). The data were further subjected to Tukey posthoc test and values at p < 0.05 were considered statistically significant. The statistical analysis was carried out using GraphPad Prism 5.03 (GraphPad Software Inc., CA, USA).

## Results

### 

#### Percentage Yield (%) of AEASAB

For the two extraction processes of AEASAB (n = 2), the percentage yield that was obtained was 66.03 ± 0.23 % (Table 2).

**Table 2:** Percentage Yield (%) of AEASAB

	Weight of Fresh Garlic Bulbs (g)	Yield of AEASAB (g)	Percentage Yield (%) of AEASAB
1st Extraction Process	800.00	526.40	65.80

2nd Extraction Process	800.00	530.08	66.26

The percentage (%) yield of AEASAB that was obtained = 66.03 ± 0.23 % (n = 2).

#### Qualitative and Quantitative Phytochemical Screening

With the exception of terpenoids that were absent, the following phytochemicals were present in gramme per 100 g of *Allium sativum* (Linn.) bulbs aqueous extract of; saponins (0.30 ± 0.03), tannins (2.95 ± 0.10), flavonoids (0.08 ± 0.02), alkaloids (0.13 ± 0.03), and cardiac glycosides (2.03 ± 0.20). This is as depicted in Table 3.

**Table 3:** Qualitative and Quantitative Phytochemical Screenings of Aqueous Extract of *Allium sativum* (Linn.)

Phytochemicals	Status	Percentage Composition (g/100g)
Saponins	+	0.30 ± 0.03
Terpenoids	_	nil
Tannins	+	2.95 ± 0.10
Flavonoids	+	0.08 ± 0.02
Alkaloids	+	0.13 ± 0.03
Cardiac Glycosides	+	2.03 ± 0.20

Each values (n = 3) is expressed as mean ± Standard Error of Mean; + = present; - = absent.

#### Oral LD_50_ of AEASAB

The oral lethal dose of AEASAB was determined to be > 2800 mg/kg in adult Wistar rats. This is because no mortality was recorded at doses up to 2800 mg/kg of oral AEASAB administration (Table 4).

**Table 4:** Oral Toxicity Test (LD50) of AEASAB

1st PHASE			
No. of rats	Dose (mg/kg)	Mortality
5	5	0/3
5	50	0/3
3	500	0/3

2nd PHASE		
No. of rats	Dose (mg/kg)	Mortality
2	350	0/2
2	700	0/2
2	1400	0/2
2	2800	0/2

→ Oral LD_50_ of AEASAB > 2800 mg/kg in adult Wistar rats.

#### Relative Testicular Weight [RTW (%)], Relative Brain Weight [RBW (%)] and Percentage Weight Change [PWC (%)]

There was a significant decrease in the RTW in groups 2, 3, 4 and 5 (0.79 ± 0.05, 0.85 ± 0.03, 0.99 ± 0.04 and 1.04 ± 0.03, respectively) when compared with that of the control (1.24 ± 0.04) (F = 20.71, p < 0.0001). A significant increase was recorded in groups 4, 5, 6 and 7 (0.99 ± 0.04, 1.04 ± 0.03, 1.15 ± 0.08 and 1.16 ± 0.05, respectively) when compared with Pb group (0.79 ± 0.05) (F = 8.140, p = 0.0005) and Pb + recovery group (0.85 ± 0.03) (F = 6.614, p = 0.0015).

At the end of the study period, groups 6 and 7 (1.15 ± 0.08 and 1.16 ± 0.05, respectively) recorded a significant increase in RTW when compared with Pb + recovery group (0.85 ± 0.03) (F = 9.500, p = 0.0034) and Pb + vitamin E group (0.99 ± 0.04) (F = 4.600, p = 0.0413) (Table 5).

**Table 5:** Effects of AEASAB on the Relative Testicular Weight (RTW), Relative Brain Weight and Percentage Weight Change (%) of Wistar Rats with Pb-Induced Reproductive Toxicity

Parameters	Groups (n = 5)						

	(1) Control	(2) Pb only	(3) Pb + Recovery	(4) Pb + Vitamin E	(5) Pb + 50 mg/kg AEASAB	(6) Pb + 100 mg/kg AEASAB	(7) Pb + 200 mg/kg AEASAB
RTW (%)	1.24±0.04	0.79±0.05*	0.85±0.03*	0.99±0.04*^§α^	1.04±0.03*^§α^	1.15±0.08^§αμ^	1.16±0.05^§αμ^
RBW (%)	0.88±0.03	0.61±0.02*	0.69±0.03*^§^	0.77±0.06*^§α^	0.74±0.03*^§^	0.79±0.05*^§α^	0.81±0.04^§α^
Percentage Weight Change (%)	22.70±1.27	2.13±1.21*	14.48±1.52*^§^	18.53±1.95*^§α^	20.01±2.64^§α^	20.21±1.59^§α^	21.03±0.40^§α^

Each value represents mean ± S.E.M (n = 5) at p < 0.05

* = significantly different from control group;

§ = significantly different from Pb group;

α = significantly different from Pb + Recovery group

μ = significantly different from Pb + Vitamin E group.

Exposure to Pb toxicity induced a significant decrease in the RBW in groups 2, 3, 4, 5 and 6 (0.61 ± 0.02, 0.69 ± 0.03, 0.77 ± 0.06, 0.74 ± 0.03 and 0.79 ± 0.05, respectively) when compared with the control (0.88 ± 0.03) (F = 5.496, p = 0.0016). This was significantly increased in groups 3, 4, 5, 6 and 7 (0.69 ± 0.03, 0.77 ± 0.06, 0.74 ± 0.03, 0.79 ± 0.05 and 0.81 ± 0.04, respectively) when compared with the Pb group (0.61 ± 0.02) (F = 3.339, p = 0.0198). Groups 4, 6 and 7 (0.77 ± 0.06, 0.79 ± 0.05 and 0.81 ± 0.04, respectively) recorded a significant increase when compared with Pb + recovery group (0.69 ± 0.03) (F = 4.287, p = 0.0313) (Table 5).

Groups 2, 3 and 4 (2.13 ± 1.21, 14.48 ± 1.52 and 18.53 ± 1.95, respectively) recorded a significant decrease in PWC when compared with the control (22.70 ± 1.27) (F = 34.31, p < 0.0001). A significant increase was recorded in groups 4, 5, 6 and 7 (18.53 ± 1.95, 20.01 ± 2.64, 20.21 ± 1.59 and21.03 ± 0.40, respectively) when compared with Pb group (2.13 ± 1.21) (F = 21.54, p < 0.0001) and Pb + recovery group (14.48 ± 1.52) (F = 9.215, p = 0.0112). Pb + recovery group (14.48 ± 1.52) recorded a significant increase in PWC when compared with Pb group (2.13 ± 1.21) (F = 1.578, p = 0.0002) (Table 5).

#### Sperm Counts [SC (million/ml)], Sperm Motility [SM (%)] and Sperm Viability [SV (%)]

SC was significantly reduced in both the Pb group (45.80 ± 4.61) and Pb + recovery group (58.40 ± 4.87) when compared with the control group (86.20 ± 5.12) (F = 18.01, p = 0.0002). This was significantly reversed in the AEASAB-treated groups 5, 6 and 7 (75.00 ± 3.66, 77.00 ± 2.84 and 80.80 ± 3.48, respectively) when compared with the Pb group (45.80 ± 4.61) (F = 18.87, p < 0.0001). At the end of the study, it was recorded that group 7 (80.80 ± 3.48) recorded a significant increase in SC when compared with the Pb + recovery group (58.40 ± 4.87) (F = 3.856, p = 0.0034) (Table 6).

**Table 6:** Effects of AEASAB Treatment on Sperm Characteristics in Wistar Rats with Pb-Induced Reproductive Toxicity

Parameters	Groups (n = 5)						

	(1) Control	(2) Pb only	(3) Pb + Recovery	(4) Pb + Vitamin E	(5) Pb + 50 mg/kg AEASAB	(6) Pb + 100 mg/kg AEASAB	(7) Pb + 200 mg/kg AEASAB
Sperm Counts (million/ml)	86.20 ± 5.12	45.80 ± 4.61*	58.40 ± 4.87*	73.20 ± 5.68^§^	75.00 ± 3.66^§^	77.00 ± 2.84^§^	80.80 ± 3.48^§α^
Sperm Motility (%)	70.00 ± 3.16	46.00 ± 5.09*	54.00 ± 4.00*	67.00 ± 3.74^§^	66.00 ± 2.45^§^	70.00 ± 3.16^§^	67.00 ± 3.12^§^
Sperm Viability (%)	86.60 ± 1.99	57.80 ± 2.65*	69.80 ± 2.54*^§^	79.40 ± 2.05^§α^	79.60 ± 2.02^§α^	80.40 ± 1.33^§α^	83.60 ± 1.35^§α^

Each value represents mean ± S.E.M (n=5) at p< 0.05.

* = significantly different from control group;

§ = significantly different from Pb group;

α = significantly different from Pb + Recovery group.

A significant decrease in SM was recorded in the Pb group (46.00 ± 5.09) and Pb + recovery group (54.00 ± 4.00) when compared with that of the control group (70.00 ± 3.16) (F = 7.059, p = 0.0031). When compared with the Pb group (46.00 ± 5.09), a significant increase was recorded in the AEASAB-treated groups 5, 6 and 7 (66.00 ± 2.45, 70.00 ± 3.16 and 67.00 ± 3.12, respectively) (F = 9.316, p = 0.0008) (Table 6).

Although the SV was significantly reduced in the Pb group (57.80 ± 2.65) when compared with the control group (86.60 ± 1.99) (F = 1.773, p < 0.0001), a significant t increase in SV was recorded in the AEASAB-treated groups 5, 6 and 7 (79.60 ± 2.02, 80.40 ± 1.33 and 83.60 ± 1.35, respectively) when compared with both Pb group (57.80 ± 2.65) (F = 38.08, p < 0.0001) and Pb + recovery group (69.80 ± 2.54) (F = 10.05, p = 0.0006). At the end of the study, the Pb + recovery group recorded a significant decrease in SV (69.80 ± 2.54) when compared with that of the control group (86.60 ± 1.99) (F = 1.629, p = 0.0008) (Table 6).

#### Levels of Reduced Glutathione (GSH) in the Brain (μg/mg protein) and Testis (μg/mg protein)

The brain level of GSH was significantly reduced in groups 2, 3 and 4 (1.25 ± 0.16, 1.59 ± 0.16 and 2.19 ± 0.18, respectively) when compared with that of the control group (2.62 ± 0.14) (F = 14.48, p < 0.0001).

**Figure 1:**
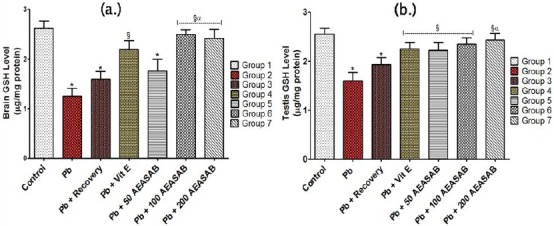
Effects of AEASAB on Levels of GSH in the Brain (a.) and Testis (b.) of Wistar Rats with Pb-Induced Reproductive Toxicity. Each value represents mean ± S.E.M (n = 5) at p < 0.05. * = significantly different from control group; § = significantly different from Pb group; and α = significantly different from Pb + Recovery group.

AEASAB treatment significantly reversed this alteration in groups 6 and 7 (2.49 ± 0.10 and 2.42 ± 0.18, respectively) when compared with Pb group (1.25 ± 0.16) (F = 21.41, p = 0.0001) and Pb + recovery group (1.59 ± 0.16) (F = 11.06, p = 0.0019) (Figure 1a).

A significant decrease in the GSH levels in the testis of the rats was recorded in groups 2 and 3 (1.60 ± 0.17 and 1.93 ± 0.14, respectively) when compared with that of the control group (2.55 ± 0.12)

#### Levels of thiobarbituric acid reactive substances (TBARS) in the Brain (nmol/mg protein) and Testis (nmol/mg protein)

**Figure 2:**
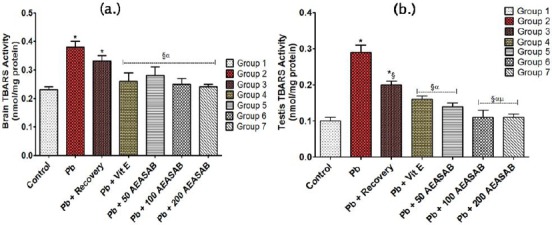
Effects of AEASAB on Levels of TBARS in the Brain (a.) and Testis (b.) of Wistar Rats with Pb-Induced Reproductive Toxicity. Each value represents mean ± S.E.M (n = 5) at p < 0.05. * = significantly different from control group; § = significantly different from Pb group; α = significantly different from Pb + Recovery group; and μ = significantly different from Pb + Vitamin E group.

The study recorded a significant increase in the levels of TBARS in the brain of the rats in the Pb group (0.38 ± 0.02) and Pb + recovery group (0.33 ± 0.02) when compared with that of the control group (0.23 ± 0.01) (F = 19.44, p = 0.0002). However, the AEASAB-treated groups 5, 6 and 7 (0.28 ± 0.03, 0.25 ± 0.02 and 0.24 ± 0.01, respectively) recorded a significant decrease in TBARS levels when compared with both the Pb group (0.38 ± 0.02) (F = 9.093, p = 0.0010) and Pb + recovery group (0.33 ± 0.02) (F = 3.630, p = 0.0359) (Figure 2a).

In the testes of the rats, there was a significant increase in TBARS level in the Pb group (0.29 ± 0.02) as well as Pb + recovery group (0.20 ± 0.01) when compared with the control group (0.10 ± 0.01) (F = 40.50, p < 0.0001). Also, a significant decrease in TBARS level was recorded in the AEASAB-treated groups 5, 6 and 7 (0.14 ± 0.01, 0.12 ± 0.02 and 0.12 ± 0.01, respectively) when compared with that of the Pb group (0.29 ± 0.02) (F = 27.03, p < 0.0001). It is worthy of note to state that groups 6 and 7 (0.11 ± 0.02 and 0.11 ± 0.01, respectively) both recorded a significant decrease in TBARS level of the testes when compared with the Pb + Vitamin E group (0.16 ± 0.01) (F = 1.000, p = 0.0077) (Figure 2b).

#### Plasma Levels of Follicle Stimulating Hormone [FSH (mIU/ml)], Luteinizing Hormone [LH (mIU/ml)] and Testosterone (ng/ml)

The plasma level of FSH was significantly reduced in the Pb group (0.13 ± 0.01) and Pb + recovery group (0.17 ± 0.01) when compared with the control (0.25 ± 0.01) (F = 37.33, p < 0.0001). Following AEASAB administration, groups 5, 6 and 7 (0.21 ± 0.01, 0.22 ± 0.02 and 0.24 ± 0.02, respectively) recorded a significant increase in the plasma level of FSH when compared with the Pb group (0.13 ± 0.01) (F = 9.333, p = 0.0008). At the end of the study period, group 7 (0.24 ± 0.02) recorded a significant increase in plasma FSH level when compared with Pb + recovery (0.17 ± 0.01) (F = 4.000, p = 0.0140) (Table 7).

**Table 7:** Effects of AEASAB on the Plasma Levels of Follicle Stimulating Hormone (FSH), Luteinizing Hormone (LH) and Testosterone in Male Wistar Rats with Pb-Induced Reproductive Toxicity

Parameters	Groups (n = 5)						

	(1) Control	(2) Pb only	(3) Pb + Recovery	(4) Pb + Vitamin E	(5) Pb + 50 mg/kg AEASAB	(6) Pb + 100 mg/kg AEASAB	(7) Pb + 200 mg/kg AEASAB
FSH (mIU/ml)	0.25±0.01	0.13±0.01*	0.17±0.01*	0.20±0.02^§^	0.21±0.01^§^	0.22±0.02^§^	0.24±0.02^§α^
LH (mIU/ml)	0.33±0.01	0.20±0.02*	0.24±0.01*	0.29±0.02^§^	0.31±0.02^§α^	0.31±0.01^§α^	0.32±0.02^§α^
Testosterone (ng/ml)	0.45±0.01	0.24±0.02*	0.33±0.02*^§^	0.39±0.01^§α^	0.41±0.02^§α^	0.42±0.01^§α^	0.44±0.01^§αμ^

It was recorded that plasma LH level was significantly decreased in both Pb group (0.20 ± 0.02) and Pb + recovery group (0.24 ± 0.01) when compared with that of the control (0.33 ± 0.01) (F = 39.81, p < 0.0001). AEASAB-treated groups 5, 6 and 7 (0.31 ± 0.02, 0.31 ± 0.01 and 0.32 ± 0.02, respectively) recorded a significant increase when compared with Pb group (0.20 ± 0.02) (F = 9.949, p = 0.0006) and Pb + recovery group (0.24 ± 0.01) (F = 5.467, p = 0.0088) (Table 7).

Plasma testosterone level was significantly decreased in the Pb group (0.24 ± 0.02) as well as Pb + recovery group (0.33 ± 0.02) when compared with that of the control (0.45 ± 0.01) (F = 37.00, p < 0.0001). Following AEASAB treatment, groups 5, 6 and 7 recorded a significant increase (0.41 ± 0.02, 0.42 ± 0.01 and 0.44 ± 0.01, respectively) when compared with both the Pb group (0.24 ± 0.02) (F = 34.23, p < 0.0001) and Pb + recovery group (0.33±0.02) (F = 9.333, p = 0.0008). Group 7 recorded a significant increase (0.44 ± 0.01) in plasma level of testosterone when compared with the Pb + vitamin E group (0.39 ± 0.01) (F = 1.000, p = 0.0077) (Table 7).

#### Histopathological Examination - Pituitary

**Figure 3:**
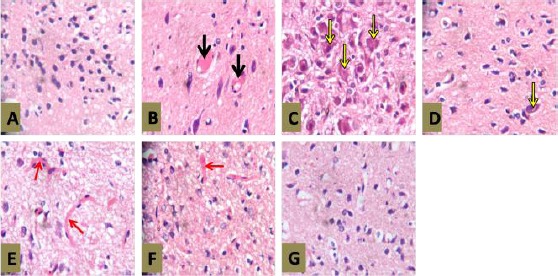
*Photomicrographs of Pituitary gland with AEASAB Treatment in Rats with Pb-Induced Reproductive Toxicity. H & E Stain (Mg. ×100). Photomicrographs of the pituitary tissue showing the effects of AEASAB administration in rats exposed to Pb toxicity. A = Control group (1); B = Pb group (2); C = Pb + recovery group (3); D = Pb + vitamin E group (4); E = Pb + 50 mg AEASAB (group 5); F = Pb + 100 mg AEASAB (group 6); and G = Pb + 200 mg AEASAB (group 7)*.

Photomicrographs of the pituitary of rats in group 2 (Figure 3B) showed evidence of vascular congestion (black arrow). This congestion appears milder (red arrow) in groups 5 and 6 (Figures 3E and 3F) with a relative improvement in the histoarchitecture of the pituitary.

Group 7 (Figure 3G) appears to be similar to group 1 (Figure 3A), but for the presence of mild vacuolation. Group 4 (Figure 3D) also show evidence of mild vacuolation. There is evidence of hypertrophic and abnormal cells (Yellow arrow) in group 3 (Figure 3C). Cells of groups 2 and 5 appear fewer (Figures 3B and 3E).

#### Histopathological Examination - Testis

**Figure 4:**
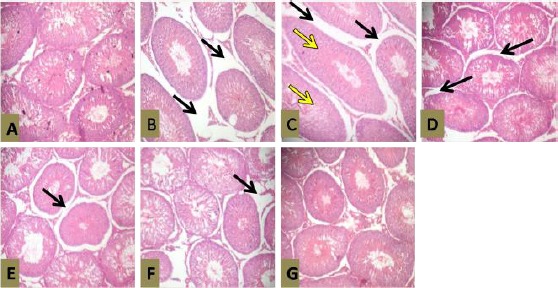
*Photomicrographs of Testis with AEASAB Treatment in Rats with Pb-Induced Reproductive Toxicity. H & E Stain (Mag. ×100). Photomicrographs of the testis showing effects of AEASAB administration in rats exposed to Pb toxicity. A = Control group (1); B = Pb group (2); C = Pb + recovery group (3); D = Pb + vitamin E group (4); E = Pb + 50 mg AEASAB (group 5); F = Pb + 100 mg AEASAB (group 6); and G = Pb + 200 mg AEASAB (group 7)*.

Photomicrographs of the rats’ testes in the different groups showing severe vacuolization of the testicular histoarchitecture (Black arrow). This vacuolization appears to be relatively milder in groups 5 and 6 (Figures 4E and 4F). There is evidence of distorted seminiferous tubule with elongated features (Yellow arrow) in groups 2 and 3 (Figures 4B and 4C). Groups 4 and 7 (Figures 4D and 4G) appear similar to group 1 (Figure 4A), but with evidence of mild vacuolation.

## Discussion

The study investigated the effects of administration of aqueous extract of *Allium sativum* (Linn.) bulbs (AEASAB) on the pituitary and testis of rats that were exposed to lead (Pb) toxicity. There was a comparative assessment of reproductive function in rats in the AEASAB-treated groups and the group that was treated with vitamin E after Pb-induced compromise to reproductive function.

According to existing literature, there seems to be apparently conclusive evidence that Pb intoxication is associated with a decrease in the body weight of exposed subjects. The significant decrease in body weight associated with Pb administration that was recorded is consistent with the findings of Aziza *et al* [42], Nadia *et al* [43] and Amjad *et al* [44]. Physically, the rats were observed to manifest signs of weakness during the period of Pb administration. This lethargy was observed to be accompanied by loss of appetite, as a large amount of the feed were untouched during this period. The significant decrease in the body weight that was observed during this period may be attributed to a decline in food consumption since weight change depends on a balance between energy expenditure and food consumption. The recovery of the weight loss in the AEASAB-treated groups could mean that AEASAB administration is associated with weight gain in a rat model, probably by promoting a healthy appetite. The precise mechanism by which AEASAB achieves this result is subject to further investigation. Although studies have shown that no single phytochemical can be considered an effective weight control product [45], it is possible that certain of the extract phytochemicals possesses rewarding effects due to their ability to stimulate an increase in food intake like that expressed by cannabinoids and *Sutherlandia* leaf powder [45]. This is also worthy of further investigation.

Ani *et al* [46] as well as Smith and Cass [47], reported that the brain is susceptible to the neurotoxic effect of Pb. The photomicrographs of the pituitary showed marked vascular congestions and evidence of fewer pituitary cells (which could be non-exclusive to this region) during the period of Pb intoxication. This could possibly be the explanation for the significant decrease in the Relative Brain Weight (RBW) that was recorded. Similar findings on the reduction in Relative Testicular Weight (RTW) of rats that were exposed to Pb toxicity, as recorded in this study, have been reported by Ahmed *et al*. [48], Elias *et al* [49], Lu *et al* [50], and Nadia *et al* [51]. The mass of differentiated spermatogenic cells is the principal determinant of testis weight [52]. Hence, the recorded significant decrease in RTW could be a result of decreased number of germ cells resulting from the observed severe vacuolization of the testicular interstitium. This was corroborated by a significant decline in plasma testosterone levels; the hormone responsible for differentiation of male sexual characteristics [53]. The study, therefore, suggests that severe vascular congestion and a reduced number of cells in the brain, as well as testicular interstitial vacuolation in the testis, is associated with a Pb-induced decrease in RBW and RTW respectively, of exposed subject. The administration of AEASAB to Pb-treated rats was able to significantly restore the weight loss in both the brain and testes in a dose-dependent manner. This may have been affected via the recorded enhancement of testosterone secretion by the Leydig’s cells in the testicular interstitium. The growth, development and maintenance of male reproductive organs require the availability of testosterone in the circulation [53]. This ameliorative potential demonstrated that AEASAB possesses anti-vacuolating and testosterone-boosting potentials in rat model exposed to Pb toxicity. This potential may have been enhanced by the presence of alkaloids and tannins in the extract. Alkaloids are reputed to have analgesic, adaptogenic and anti-inflammatory effects which help the host (animal or man) to develop endurance against stress and resistance against disease [54] while tannins hasten the healing of wounds and inflamed mucous membrane [55, 56]. These important potentials of the extract’s phytochemicals are essential for reversing the deleterious effects of xenobiotics (like lead) on reproductive functions.

GSH is probably the most important antioxidant present in body cells [57]. This is because it effectively removes hydrogen peroxide and serves as a cofactor for glutathione transferase, which helps to remove certain drugs, chemicals and other reactive molecules from the cells [57]. The significant decrease in GSH levels of rats that was observed in this study could be as a result of excessive use of GSH by the tissues to scavenge free radicals that were generated as a result of exposure to Pb toxicity and/or decreased the production of GSH by the tissues following Pb intoxication. The significant increase in TBARS that was recorded in the tissues indicated high levels of lipid peroxidation following exposure to Pb toxicity. The result of this study indicated that AEASAB administration was able to attenuate the deleterious alterations in the levels of GSH and TBARS that were induced by Pb intoxication, in a dose-dependent manner. This may be as a result of its ability to scavenge free radicals and/or enhance homeostasis in the antioxidant system in rat model by enhancing the GSH-producing capacity of the tissues. Therefore, the study demonstrated the ability of AEASAB to restore antioxidant status in vivo; since the reduction of antioxidant activity may be due to accumulation of free radicals (generated by certain chemicals and heavy metals like Pb), enhancement of lipid peroxidation and or inactivation of the antioxidant system [58]. The antioxidant property of the extract can be attributed to the presence of flavonoids and tannins; which are reputed to have high antioxidant activity [59, 60], inhibit tumor formation and decrease inflammation [61]. It is worthy of note to state that the AEASAB-treated groups at higher doses produced a better-attenuating profile of lipid peroxidation level in the testis of the rats when compared with the group that received vitamin E treatment.

The cumulative result of all the stages of sperm production is provided by sperm count analysis. This makes it one of the most reliable tests for spermatogenesis [62]. The fact that it is highly correlated with fertility makes it one of the most sensitive tests for spermatogenesis [63]. The Pb-induced reduction in sperm count is indicative of the fact that reproductive function was compromised. The Pb-induced decrease in sperm motility that was recorded may be both functional and structural in origin. The decline in GSH levels with a deleterious increase in TBARS levels in the testes, as recorded in this study, supports this fact. These indices show evidence of assault to the reproductive tissues. This fact is corroborated by the severe vacuolation in the testicular tissues that was observed in the representative photomicrographs. Another possible explanation for the significant decrease in sperm motility may be the ability of Pb to permeate the blood-testis barrier; this may be a possible explanation for the corresponding decrease in sperm viability that was observed during the period of Pb intoxication. The pH and osmolarity of semen specimen are important factors that affect sperm viability [64]. A significant decrease in sperm motility (and viability) may occur if chemical agents permeate the blood-testis barrier. This creates a different microenvironment in the inner aspects of the seminiferous tubules when compared with its outer parts [58], with a consequent compromise to reproductive functions. Pb administration induced deleterious alterations in the testicular tissues which were characterized by severe vacuolization. In a dose-dependent manner, AEASAB administration was able to reverse the Pb-induced alterations in sperm characteristics that were observed. This was recorded to be in a relatively higher degree when compared with the group that received treatment with vitamin E. Also, the antioxidant status of the AEASAB-treated groups (demonstrated by GSH and TBARS levels) was reinstated towards normal values in a dose-dependent fashion at the end of the study. Based on the result of this study, the suggested mechanism of action of AEASAB in the reversal of Pb-induced alterations in sperm characteristics could be in its antioxidant and membrane-stabilizing potentials; enhanced by the presence of phytochemicals such as tannins, alkaloids and flavonoids in its extract [59-61]. The study demonstrated that the activities of these important phytochemicals have secondary beneficial effects on the restoration of normal reproductive function in male Wistar rats following Pb-induced reproductive dysfunction.

The two independent but synchronized functions that control overall testicular function is the biosynthesis of androgens by the Leydig cells and the production of spermatozoa in the epithelium of seminiferous tubules [66]. The Pb-induced reductions in the plasma level of reproductive hormones, as recorded in this study, have been reported by Amjad *et al* [44]. The activity of FSH and LH is dependent on both the quantity of these hormones and the number of their specific receptors in the testes [67]. This study revealed features of Pb-induced vascular congestion and cellular hypertrophy in the pituitary of the rats. This explains the decreasing levels of FSH and LH that were recorded in this study. There may have been decreasing secretion of these hormones as a consequence of this histopathology, reduced sensitivity and or inadequate specific receptors for these hormones in the testes. This may be accompanied by a reduction in Leydig cell steroidogenesis, as reported by [68] and [69]. Spermatogenesis is initiated by the presence of FSH and it is maintained by testosterone secretion from the Leydig cells. LH from the pituitary is, however, essential for the secretion of testosterone [67]. The recorded significant decrease in testosterone secretion could have been as a result of both decreasing level of LH secretion by the pituitary and pathological changes that may have occurred in the Leydig cells that are present in the interstitial tissues. This study revealed that AEASAB administration produced a dose-dependent ameliorative effect on Pb-induced alterations in the gonadotropin levels of rat model. These (ameliorative) effects were found to be higher (but not significantly) when compared with the group that received vitamin E treatment. This suggests that AEASAB has a better health profile on reproductive function with increased doses. The activity of AEASAB was reflected in the reversal of RTW loss, as well as remarkable attenuation of the deleterious derangements in plasma levels of FSH, LH and testosterone. The study, therefore, suggests that AEASAB administration has the potential to restore gonadotropin homeostasis in rats that are exposed to Pb toxicity, via structural and functional repair of the pituitary and testes in a rat model. Although there is a dearth of literature on the effects of the extract’s phytochemicals on serum gonadotropin level, the study demonstrated that the extract is rich in phytochemicals that enhances the restoration of Pb-induced alterations in gonadotropin homeostasis. The precise phytochemical(s) that is responsible for this action is worthy of further investigation.

Based on the overall data gathered in this study, it was demonstrated that vitamin E showed a relatively lower capacity to ameliorate the Pb-induced deleterious alterations in reproductive function at 100 mg/kg/day (a dose apparently accepted to be an established therapeutic dose in rat model [35-37]) when compared with the AEASAB-treated groups. It is, therefore, suggested that this accepted dose be reviewed, otherwise, the therapeutic administration of vitamin E alone may not be sufficient to produce the desired ameliorative features in a rat model with Pb-induced reproductive toxicity.

In conclusion, the administration of AEASAB ameliorated pituitary-testicular injury and dysfunction in rats with Pb-induced reproductive disturbances. This expressed potential may be attributed to its antioxidant and membrane stabilizing effects on the pituitary and testis to bring about homeostasis restoration in reproductive function. Therefore, aqueous extract of *Allium sativum* bulbs represents a prospective therapeutic choice to ameliorate Pb-induced reproductive disturbances and dysfunctions in exposed subjects.
